# A Novel Agarase, Gaa16B, Isolated from the Marine Bacterium *Gilvimarinus agarilyticus* JEA5, and the Moisturizing Effect of Its Partial Hydrolysis Products

**DOI:** 10.3390/md20010002

**Published:** 2021-12-21

**Authors:** Youngdeuk Lee, Eunyoung Jo, Yeon-Ju Lee, Tae-Yang Eom, Yehui Gang, Yoon-Hyeok Kang, Svini Dileepa Marasinghe, Sachithra Amarin Hettiarachchi, Do-Hyung Kang, Chulhong Oh

**Affiliations:** 1 Jeju Marine Research Center, Korea Institute of Ocean Science and Technology, Gujwa-eup, Jeju 63349, Korea; lyd1981@kiost.ac.kr (Y.L.); jey8574@kiost.ac.kr (E.J.); eomsun@kiost.ac.kr (T.-Y.E.); yehui@kiost.ac.kr (Y.G.); yoon-hyeok@kiost.ac.kr (Y.-H.K.); svini91@kiost.ac.kr (S.D.M.); amarin@kiost.ac.kr (S.A.H.); dohkang@kiost.ac.kr (D.-H.K.); 2 Marine Natural Products Chemistry Laboratory, Korea Institute of Ocean Science and Technology, 385 Haeyangro, Busan 49111, Korea; yjlee@kiost.ac.kr; 3 Department of Ocean Science, University of Science and Technology, 217 Gajeong-ro, Yuseong-gu, Daejeon Metropolitan City 34113, Korea; 4 Department of Fisheries and Aquaculture, Faculty of Fisheries and Marine Sciences & Technology, University of Ruhuna, Matara 81000, Sri Lanka

**Keywords:** β-agarase, glycoside hydrolase family 16, neoagaro-oligosaccharide, partial hydrolytic product, hyaluronidase inhibition, *Gilvimarinus agarilyticus* JEA5

## Abstract

We recently identified a β-agarase, Gaa16B, in the marine bacterium *Gilvimarinus agarilyticus* JEA5. Gaa16B, belonging to the glycoside hydrolase 16 family of β-agarases, shows less than 70.9% amino acid similarity with previously characterized agarases. Recombinant Gaa16B lacking the carbohydrate-binding region (rGaa16Bc) was overexpressed in *Escherichia coli* and purified. Activity assays revealed the optimal temperature and pH of rGaa16Bc to be 55 °C and pH 6–7, respectively, and the protein was highly stable at 55 °C for 90 min. Additionally, rGaa16Bc activity was strongly enhanced (2.3-fold) in the presence of 2.5 mM MnCl_2_. The *K_m_* and *V_max_* of rGaa16Bc for agarose were 6.4 mg/mL and 953 U/mg, respectively. Thin-layer chromatography analysis revealed that rGaa16Bc can hydrolyze agarose into neoagarotetraose and neoagarobiose. Partial hydrolysis products (PHPs) of rGaa16Bc had an average molecular weight of 88–102 kDa and exhibited > 60% hyaluronidase inhibition activity at a concentration of 1 mg/mL, whereas the completely hydrolyzed product (CHP) showed no hyaluronidase at the same concentration. The biochemical properties of Gaa16B suggest that it could be useful for producing functional neoagaro-oligosaccharides. Additionally, the PHP of rGaa16Bc may be useful in promoting its utilization, which is limited due to the gel strength of agar.

## 1. Introduction

Agar is the main component of the cell wall in several species of red seaweed and consists of a mixture of two polysaccharides: agarose and agaropectin. Agarose consists of a linear chain of alternating 3-O-linked β-d-galactopyranose and 4-O-linked 3,6-anhydro-α-l-galactose units [1]. Agar is widely used as a gelling substance in the pharmaceutical, cosmetic, and food industries, as well as in the life sciences, where it is used in a range of techniques, including microbiome culturing media, electrophoresis gel, and chromatography resin. In addition to these classical applications, many agar-derived oligosaccharides exhibit various biological and therapeutic properties and, thus, have the potential for diverse applications in the cosmetic, medicinal, and pharmaceutical industries [2,3].

Agarases hydrolyze both agar and agarose. Based on the mechanism of their actions, agarases are categorized into two main groups, namely, α-agarases and β-agarases. α-agarases hydrolyze α-1,3-linkages between the composite sugars in agarose to generate agaro-oligosaccharides (AOS), whereas β-agarases hydrolyze only β-1,4-linkages to generate neoagaro-oligosaccharides (NAOS). β-agarases are classified into four families based on their amino acid sequence homology: glycoside hydrolase 16 (GH16), GH50, GH86, and GH118 [4]. To date, many β-agarases have been found in various genera, including *Agarivorans* [5], *Alteromonas* [6], *Bacillus* [7], *Catenovulum* [8], *Pseudoalteromonas* [9], *Pseudomonas* [10], *Vibrio* [11], *Streptomyces* [12], *Saccharophagus* [13], and *Gilvimarinus* [14].

Among the many agarases reported, most are β-agarases, except some α-agarases isolated from *Alteromonas agarilyticus* GJ1B [15], *Thalassomonas agarivorans* JAMB-A33, *Catenovulum agarivorans* [16], and *Caternovulum sediminis* WS1-A [17]. Several reports have described that β-agarases hydrolyze agarose to produce various neoagaro-oligosaccharides, such as neoagarobiose, neoagarotetraose, and neoagarohexaose [1].

NAOS have potential for use as cosmetic materials. NAOS have been observed to exhibit high inhibition effects on tyrosinase and melanin biosynthesis [18,19]. In the cosmetics industry, the demand for products that can control skin aging is increasing [20]. Skin aging is associated with loss of skin moisture. Hyaluronic acid is a well-known key molecule involved in skin aging. It is very important to maintain the homeostasis of hyaluronic acid because it can be depolymerized by hyaluronidase; thus, inhibitors of hyaluronidase can be useful in preventing the aging of skin. However, to the best of our knowledge, the hyaluronidase inhibition of polysaccharide-derived agar has not yet been reported.

In this study, we discovered that *gaa16b,* a gene isolated from the marine bacterium *Gilvimarinus agarilyticus* JEA5, encodes a novel β-agarase. We cloned the *gaa16b* gene and overexpressed it as a recombinant protein in *E. coli* BL21 (DE3). Next, maltose-binding protein (MBP)-tagged Gaa16B was purified and analyzed to investigate its biochemical properties and hydrolytic patterns. Additionally, to validate the potential of the hydrolytic product of rGaa16Bc for cosmetic use, we carried out a hyaluronidase inhibitory assay.

## 2. Results

### 2.1. Molecular Characteristics

Previously, we published a draft genome of the marine bacterium *Gilvimarinus agarilyticus* JEA5, exhibiting agarolytic activity [21], and identified Gaa16B as a predicted agarase. The nucleotide sequence of *gaa16b* has an open reading frame (ORF) of 1800 bp, encoding a putative agarase of 600 amino acid residues. The molecular mass and isoelectric point (pI) of Gaa16B were predicted to be 65 kDa and 4.2, respectively. Analysis of the amino acid sequence predicted a signal peptide in the N-terminal region, a GH16 domain in the middle of the protein, and two carbohydrate-binding module 6 (CBM6) motifs in the C-terminus. The deduced amino acid sequence of Gaa16B showed the highest identity (90.7%) and similarity (93.8%) with *Gilvimarinus polysaccharolyticus* (WP_049721028.1), whereas it exhibited less than 55.9% identity and 70.9% similarity with functionally characterized agarases (Table 1).

### 2.2. Overexpression of Recombinant Agarase Gaa16B

**Figure 1:**
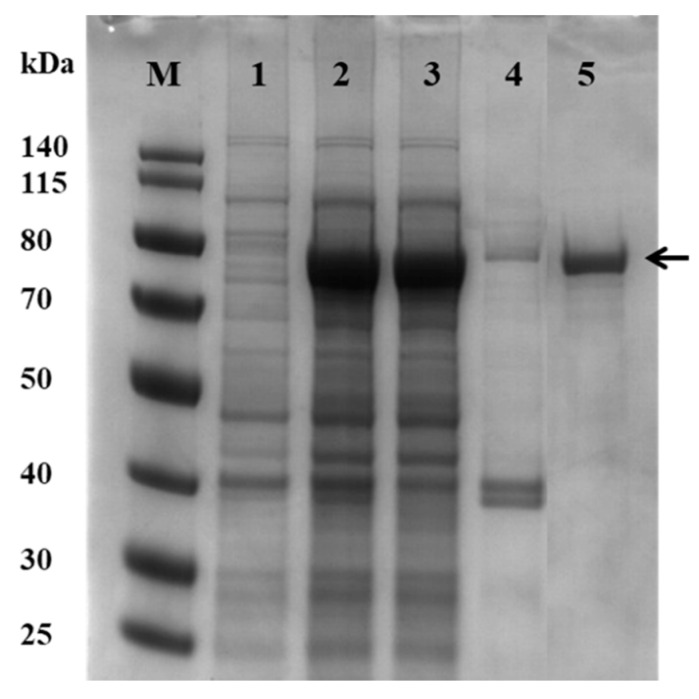
SDS-PAGE of the rGaa16Bc protein. rGaa16Bc samples were separated on a 12% SDS-PAGE gel and stained with Coomassie brilliant blue. M: Molecular mass marker (Thermo scientific, Waltham, MA, USA). Lane 1: whole cell lysates from *E. coli* BL21 (DE3) before induction; lane 2: whole cell lysates after IPTG induction; lane 3: total soluble cellular extract after induction; lane 4: total insoluble cellular extract after induction; lane 5: purified rGaa16Bc.

To characterize the Gaa16B protein, the catalytic region (the GH16 domain) of the *gaa16b* gene was amplified using PCR and cloned into the pMal-c2x expression vector. The recombinant Gaa16B catalytic domain (rGaa16Bc) fused to an MBP tag was expressed in *E. coli* BL21 (DE3) and purified using the pMal fusion protein purification system. The purified rGaa16Bc protein was electrophoresed on an SDS-PAGE gel, which exhibited a strong single band with an approximate molecular mass of 75 kDa, corresponding to the predicted molecular mass (33 kDa for rGaa16Bc plus 42 kDa for the MBP tag) (Figure 1).

### 2.3. The Optimum Conditions for rGaa16Bc Activity

**Figure 2:**
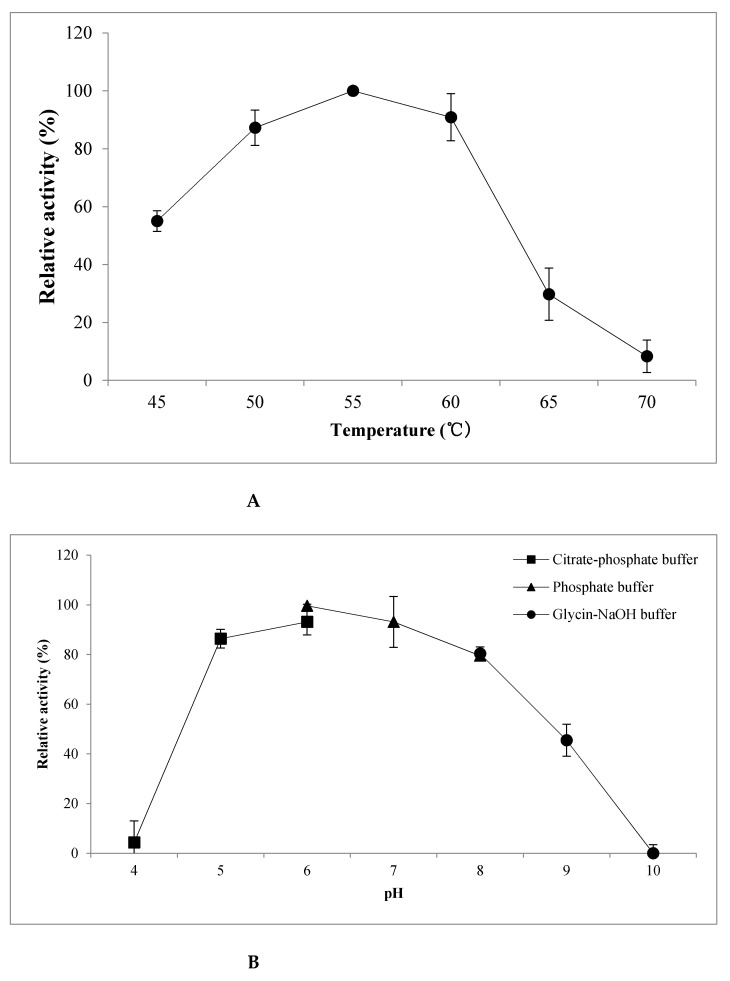

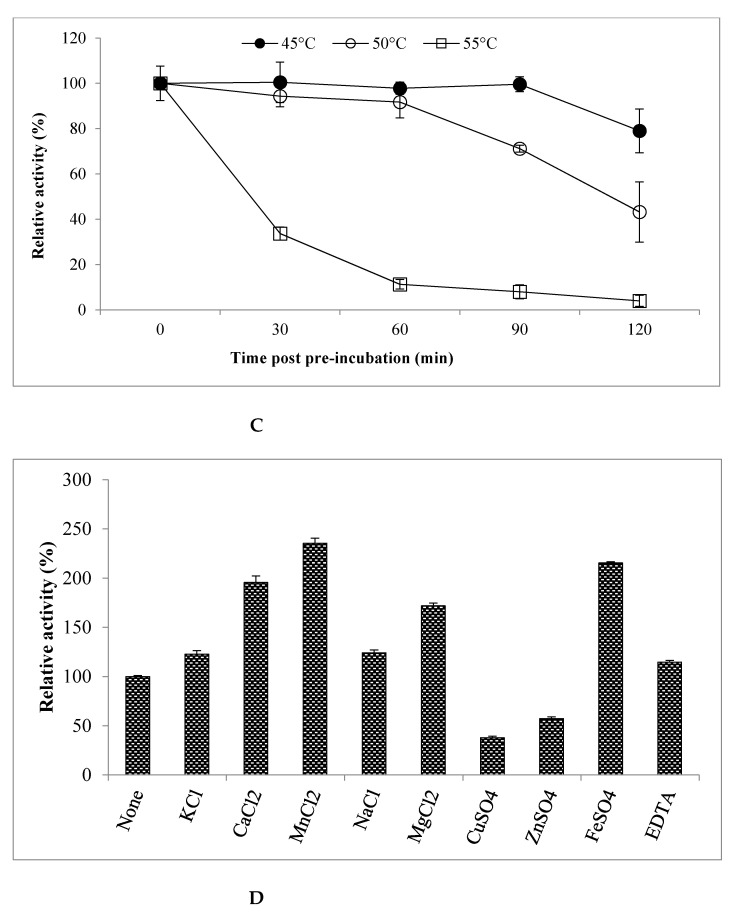
Characterization of biochemical properties of purified rGaa16Bc. The effect of temperature (**A**), pH (**B**), thermostability (**C**), and metal ions and chelators (**D**) on rGaa16Bc activity.

Next, we investigated the effects of temperature, pH, thermostability, and metal ions on rGaa16Bc activity. The recombinant protein was exposed to temperatures ranging from 45 °C to 70 °C, as shown in Figure 2A. rGaa16Bc activity gradually increased from 45 °C to 55 °C, exhibiting the highest agarolytic activity at 55 °C. Its activity dramatically decreased at temperatures over 60 °C and lost almost all activity at 70 °C. The effect of pH on rGaa16Bc activity was tested using pH values ranging from 4.0 to 10.0, in three different buffers, as shown in Figure 2B. rGaa16Bc showed over 80% activity in the pH range of 5–8, with its highest activity observed at pH 6. No activity was detected at pH 4 or 10. Although the maximum activity of rGaa16Bc was observed at 55 °C, the protein showed low thermostability at this temperature (Figure 2C). It showed less than 40% activity at 55 °C after 30 min and almost lost activity after 60 min. However, rGaa16Bc activity was stable at 45 °C for up to 90 min and maintained approximately 80% of its activity for 120 min. The rGaa16Bc protein was also very stable at 50 °C for 60 min and maintained approximately 70% of its activity for 90 min. To determine the effect of metal ions and chelators, the enzyme assay was carried out in the presence of 2.5 mM KCl, CaCl_2_, MnCl_2_, NaCl, MgCl_2_, CuSO_4_, ZnSO_4_, FeSO_4_, or EDTA (Figure 2D). The relative activity of rGaa16Bc was inhibited by CuSO_4_ and ZnSO_4_ but was enhanced in the presence of 2.5 mM MnCl_2_, CaCl_2_, MgCl_2_, and FeSO_4_. In particular, MnCl_2_ resulted in a more than twofold increase in the activity of rGaa16Bc.

The enzymatic reactions for calculating *K_m_* and *V_max_* values were performed under optimal conditions, and the results showed that the *K_m_*, *V_max_*, and *K_cat_* values of rGaa16Bc for agarose were 6.4 mg/mL, 953 U/mg, and 201.2 s^−1^, respectively.

### 2.4. Hydrolytic Pattern of rGaa16Bc

**Figure 3:**
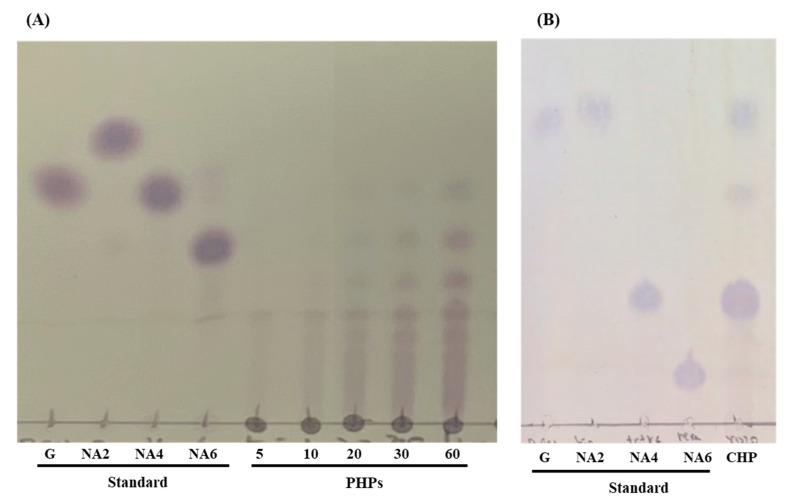
Thin-layer chromatography of PHPs (**A**) and CHP (**B**). d-galactose (G), neoagarobiose (NA2), neoagarotetraose (NA4), and neoagarohexaose (NA6) are used as standard. (**A**) The hydrolysates at different reaction time points (PHP5, PHP10, PHP20, PHP30, PHP60). (**B**) The hydrolysis product after overnight incubation (CHP).

To determine the hydrolysis pattern and products of rGaa16Bc on agar, thin-layer chromatography (TLC) was used to investigate the hydrolysates at different reaction time points. The TLC results (Figure 3) show that rGaa16Bc hydrolyzes agar, generating various neoagaro-oligosaccharides during the initial stages of the reaction. With increasing incubation time, the ratio of the larger oligosaccharides gradually decreased, while that of neoagarotetraose increased (Figure 3A). After overnight incubation, neoagarotetraose was observed to be the main product (Figure 3B).

### 2.5. Chemical Composition of PAPs

To produce PHPs composed of different ranges of molecules, a time-course hydrolysis was carried out with rGaa16Bc. The TLC results of the partial hydrolytic product are shown in Figure 3. Although the polymers of small molecules were not detected at the initial stage of the reaction, various degrees of polymers were observed over time, and oligosaccharides (less than 10 polymers) were clearly observed after 60 min.

To analyze the average molecular weight, gel permeation chromatography (GPC) experiments were performed. The results are presented in Table 2. The molecular size range of the main peak showed no significant difference in all tested samples; however, the average molecular weight (Mw) of the main peak tended to gradually decrease depending on the reaction time. The polydispersity of PHPs was 5.52~6.14, indicating a wide distribution, and there was no obvious difference between each sample. Meanwhile, the CHP that was completely hydrolyzed by the enzymatic reaction showed 1.04 of polydispersity value.

### 2.6. Hyaluronidase Inhibitory Effect of Hydrolytic Products

The effect of hyaluronidase inhibition by PHPs was also investigated. The results are presented in Table 3. The half maximal inhibitory concentration (IC_50_) was 0.47–0.57 mg/mL in all tested PHPs and showed over 60% inhibition at a concentration of 1 mg/mL. In contrast, hyaluronidase inhibition activity was hardly observed in the completely hydrolyzed sample (CHP) at a concentration of 1 mg/mL. The IC_50_ value of PHP5, which was hydrolyzed for 5 min, showed an IC_50_ value of 0.47. The IC_50_ value gradually decreased with hydrolysis time, and PHP20, which was hydrolyzed for 20 min, showed the lowest value (0.3). The IC_50_ values of PHP30 and PHP60 were 0.4 and 0.5, respectively, indicating that the IC_50_ values increased again with increasing hydrolysis time.

## 3. Discussion

We previously reported a draft genome sequence for the agar-degrading bacterium *G. agarilyticus* JEA5 [21], and we were the first to describe the molecular characteristics and biochemical properties of a β-agarase (Gaa16A) isolated from *G. agarilyticus* JEA5 [14]. Here, we describe Gaa16B, a novel neoagarotriose-producing β-agarase. This newly identified agarase possesses the typical functional domains characteristic of β-agarases belonging to the GH16 family. Almost all GH16 β-agarases contain a GH16 domain at the N-terminus and carbohydrate-binding modules in the C-terminus of the protein [22]. Gaa16B exhibits both the GH16 domain and two carbohydrate VI modules at the N- and C-termini of the protein, respectively.

The Gaa16B amino acid sequence showed the highest identity and similarity with a carbohydrate-binding protein from *G. polysacchariticus* (WP_049721028.1), which has not yet been characterized. The NCBI database contains three genomes from the *Gilvimarinus* genus; however, only one agarase (Gaa16A from *G. agarilyticus* JEA5) has been characterized to date [14]. Compared to the amino acid sequences of characterized agarases, Gaa16B displayed the highest similarity to an agarase from *Saccharophagus degradans* 2-40 (with a sequence identity and similarity of only 55.9% and 70.9%, respectively).

To determine the optimum reaction conditions for this enzyme, we investigated the effects of temperature, pH, thermostability, and metal ions on rGaa16Bc function. The highest agarolytic activity was observed at 55 °C and pH 6, although relatively high agarolytic activity (80% or more) was also observed at pH 5–8. This high activity over a relatively wide pH range may be advantageous for industrial use of rGaa16Bc. Furthermore, since agar hardens at temperatures below 40 °C, it is important to have high activity above 40 °C. rGaa16Bc showed optimum activities at these higher temperatures, as well as under neutral ionic conditions that do not require neutralization. These properties are particularly advantageous for industrial applications.

The kinetic characterization assays revealed that the *K_m_* value of rGaa16Bc (6.4 mg/mL) was only slightly higher than that of other reported GH16 β-agarases, which is probably due to the absence of the CBMs. To assess the agarolytic activity of Gaa16B, the *gaa16b* gene was amplified without the carbohydrate-binding region and cloned into a pMal-c2x vector. Most of the expressed protein was insoluble when the full-length recombinant Gaa16B (including the carbohydrate binding region) was cloned and expressed in *E. coli*, and the soluble fraction also showed very low agarolytic activity (data not shown). In contrast, rGaa16Bc without CBMs showed very high activity compared to that of full-length Gaa16B. Other studies have also reported the kinetic characterization of GH16 β-agarases lacking CBMs and reported similar findings. A recombinant agarase containing only the GH16 catalytic region from *M. thermotolerans* JAMB-A94 exhibited a higher *K_m_* value than the full-length fusion protein [23]. Additionally, Wang et al. reported that a recombinant Aga0917 lacking a CBM from *Pseudoalteromonas fuligina* YTW-15-1 showed a remarkably high *K_m_* (39.6 mg/mL) compared to other known β-agarases [24].

TLC results showed that agarose was rapidly fragmented by rGaa16Bc. In the early stages of the reaction, rGaa16Bc hydrolyzed agarose to generate neoagarobiose, neoagarotetraose, neoagarohexaose, and various larger oligosaccharides. The amount of neoagarosaccharides smaller than neoagarohexaose increased in a time-dependent manner. This hydrolytic pattern suggested that rGaa16Bc functions as an endo-type β-agarase. It has been reported that endo-type agarases randomly degrade agarose and rapidly lower the viscosity of agarose solution, while exo-type agarases tend to produce single major products and gradually decrease the viscosity of agarose solutions [10].

The bioactivity of oligosaccharides has been reported to be closely correlated with their molecular properties, such as the degree of polymerization (DP) and molecular size [25]. Kobayashi et al. reported the potential of neoagarobiose as a moisturizer by demonstrating its hygroscopic ability. Ohta et al. reported that neoagarohexaose is more effective as a skin moisturizer than smaller neoagarooligosaccharides because of its higher viscosity [26]. To obtain products in a relatively high molecular state without gel formation, we partially hydrolyzed the agar. The partially hydrolyzed products exhibited high hyaluronidase inhibitory activity, while the CHP showed no hyaluronidase inhibition activity. However, there are no reports about the high molecular weight of depolymerized agar to inhibit hyaluronidase. In this study, all tested PHPs showed over 60% hyaluronidase inhibition at a concentration of 1 mg/mL. Since hyaluronic acid has been reported to modulate skin moisture, it is easily degraded by hyaluronidase on the surface of skin [27]; a hyaluronidase inhibitor is considered a promising compound to retain skin moisture and prevent skin wrinkles. Therefore, PHPs are suggested as hyaluronidase inhibitors for the further development of cosmetic or cosmeceutical products.

Chemical hydrolysis can produce high amounts of monosaccharides with the accompanying production of undesirable toxic compounds [28]. Enzymatic hydrolysis potentially offers many advantages, such as specific hydrolysis, resulting in the production of high amounts of target oligosaccharides; is environmentally friendly; and produces low amounts of monosaccharides and toxic molecules. Despite these advantages, there is a disadvantage in that the processing cost is higher than that of chemical hydrolysis. PHPs with only 0.1 U/mL of rGaa16B showed a gradual increase in their hyaluronidase inhibition activity and exhibited the highest activity after 20 min (PHP20); however, the activity showed a tendency to decrease again after 30 min (PHP30), and no activity was detected in the final product, the CHP. Therefore, the desired product with the ability of hyaluronidase inhibition could be obtained with a small amount of enzyme and a short reaction time, thereby reducing the cost of production. This type of approach in production cost is highly advantageous, as the high cost for enzymatic hydrolysis is an inescapable factor in industry.

## 4. Materials and Methods

### 4.1. Molecular Characterization of Gaa16B

In our previous study, we isolated the marine bacterium *Gilvimarinus agarilyticus* JEA5, exhibiting agarolytic activity, and sequenced its genome using next-generation sequencing (NGS) [21]. The predicted agarase was identified by searching the draft genome sequence of *G. agarilyticus* JEA5 (NCBI accession No. WP_041522726) using the Basic Local Algorithm Search Tool (BLAST), named Gaa16B, and submitted to NCBI GenBank (NCBI accession No. KP716980). Signal peptide analysis was performed using the SignalP 4.1 server [29], and conserved domains were predicted using SMART [30] and ScanProsite [31]. The identities and similarities of the Gaa16B amino acid sequence were investigated using the EMBL pairwise sequence alignment tool [32].

### 4.2. Cloning of the gaa16b Agarase Gene

Genomic DNA was isolated from *G. agarilyticus* JEA5 (KCCM43129) using an *AccuPrep*^®^ Genomic DNA Extraction Kit (Bioneer, Daejeon, South Korea) following the manufacturer’s protocol. Primer pairs were designed to amplify only the catalytic region (GH16 domain) of Gaa16B, and the resulting protein fragment was designated as Gaa16Bc. Gaa16Bc-F (5′-TTC AGA ATT CGG ATC GCC GAC TGG GAC GGC TTA-3′) and Gaa16Bc-R (5′-TTG CCT GCA GGT CGA CTA GGT AAT GTC GTT ATC GCC ATT GT-3′) primers were designed such that the 5′ region shares 15 bp with both ends of the *BamH*I-and *SalI-digested* vector sequence. The PCR mixture consisted of 1 μL of genomic DNA template (200 ng/μL), 35.5 μL sterile deionized water, 5 μL 10X Ex Taq buffer, forward and reverse primers (20 pmol each), 4 μL dNTPs (2.5 mM), and Ex Taq DNA polymerase (3 U). PCR amplification conditions were as follows: initial denaturation at 94 °C for 5 min; 30 cycles of denaturation at 94 °C for 30 s; annealing at 58 °C for 30 s; extension at 72 °C for 1 min 30 s; and a final extension at 72 °C for 5 min. PCR reactions were conducted using a TaKaRa PCR Thermal Cycler Dice^®^ Gradient (Takara Bio Inc., Shiga, Japan). The PCR products were purified using an AccuPrep^®^ Gel Purification Kit (Bioneer, Daejeon, South Korea) and then cloned into a *BamH*I-and *SalI-digested* pMal-c2x expression vector (New England Biolabs, Hitchin, UK) using an Ez-Fusion^TM^ Cloning Kit (Enzynomics, Daejeon, South Korea) following the manufacturer’s protocol. The recombinant plasmids were transformed into *E. coli* DH5α cells using standard protocols. The clones were purified using an AccuPrep^®^ Nano-Plus Plasmid Mini Extraction Kit (Bioneer, Daejeon, South Korea) and then transformed into *E. coli* BL21 (DE3) cells for protein expression.

### 4.3. Overexpression and Purification of Recombinant Gaa16Bc

*E. coli* cells carrying Gaa16Bc-pMal-c2x were cultured overnight at 37 °C in 5 mL of Luria broth (LB) supplemented with 100 mg/mL ampicillin. From the overnight culture, 3 mL of culture was re-inoculated into 250 mL of fresh media and incubated until the mid-logarithmic phase (OD_600nm_ = 0.6–0.7). To overexpress recombinant proteins, isopropyl-β-d-thiogalactopyranoside (IPTG) was added to a final concentration of 0.05 mM, and the cultures were incubated at 20 °C for 20 h. The cells were harvested by centrifugation at 8000× *g* for 15 min. The collected cells were resuspended in a column buffer (200 mM NaCl and 20 mM Tris-HCl) and frozen at −20 °C overnight.

Frozen cells were thawed on ice and disrupted by sonication. The supernatants were separated by centrifugation at 8000× *g* for 20 min at 4 °C. Recombinant Gaa16Bc (rGaa16Bc) was purified from the soluble fraction using the pMal^TM^ Protein Fusion and Purification System (New England Biolab, UK) according to the manufacturer’s instructions and analyzed using SDS-PAGE. Gels were stained with Coomassie Brilliant Blue to visualize the proteins. The concentrations of the purified recombinant proteins were determined using a BCA protein assay reagent kit (Thermo Fisher Scientific Inc., Waltham, MA, USA).

### 4.4. Enzyme Activity Assays

To determine the biochemical properties of rGaa16Bc, we performed enzyme assays to assess the optimum temperature and pH for enzyme activity, as well as protein thermostability, and the effects of metal ions and chelators. Enzyme activities were measured using modified DNS methods [33]. The reaction conditions were as follows: 100 μL of 1% agarose (Lonza, Basel, Switzerland), 95 μL of proper buffer, and 5 μL of diluted enzyme (200 ng/uL), and incubation lasted for 5 min at 55 °C. The optimum temperature assay was conducted at 45–70 °C with 5 °C intervals. To assess the optimum pH, enzyme activity was measured from pH 4.0 to pH 10.0 at pH 1.0 intervals for 5 min at 55 °C. To analyze thermostability, rGaa16Bc was pre-incubated at 45, 50, or 55 °C for 30, 60, 90, and 120 min. Enzyme activity was measured every 30 min for 120 min. Relative activity was calculated in comparison to the maximum agarolytic activity, which was set at 100%. To determine the kinetic parameters of the recombinant protein, the agarolytic activities of rGaa16Bc were measured using various concentrations of agarose ranging from 0.25 to 10 mg/mL under optimal conditions. The *K*_m_, *V*_max_, and *K*_cat_ values were calculated using GraphPad Prism, version 8.3.1 (GraphPad Software, Inc., San Diego, CA, USA).

### 4.5. Thin Layer Chromatography (TLC)

TLC was used to analyze the hydrolytic patterns of rGaa16Bc. D-galactose (Sigma-Aldrich, Burlington, MA, USA), neoagarobiose (NA2), neoagarotetraose (NA4), and neoagarohexaose (NA6) (all from Carbosynth, Compton, Berkshire, UK) were used as standards. The hydrolytic products were applied to a silica gel 60 TLC plate (Merck, Darmstadt, Germany), which was developed using a solvent system of n-butanol: acetic acid: dH2O (2:1:1 (*v/v*)). Spots were visualized by spraying orcinol dip reagent (80 mg of orcine monohydrate was dissolved in 160 mL of acetone, to which 8 mL of sulfuric acid was added), followed by heating at 110 °C in a drying oven for 10 min.

### 4.6. Hyaluronidase Inhibitory Assay

Hydrolytic products with diverse molecular weights were prepared to evaluate their hyaluronidase inhibitory effects. Food-grade agar powder was purchased from Miryang Agar (Republic of Korea). The agar was dissolved in dH_2_O. The agar solution (1%) was treated with purified rGaa16B (final concentration of 0.1 unit/mL). Each reaction mixture was incubated at 55 °C for 5, 10, 20, 30, and 60 min and designated as partial hydrolytic products (PHP) 5, PHP10, PHP20, PHP30, and PHP60, respectively. After each reaction time, the reaction mixture was boiled at 90 °C for 10 min to inactivate the enzyme. The completely hydrolyzed product (CHP) was prepared using rGaa16Bc overnight under optimum conditions.

The PHPs, CHP, and anti-allergic drug DSCG (disodium cromoglycate, positive control) were evaluated for hyaluronidase inhibitory activity. Disodium cromoglycate (DSCG), a potent antiallergen, has a strong inhibitory effect on hyaluronidase. Kakegawa et al. reported that DSCG inhibited the activity of inactive hyaluronidase more strongly than the activated enzyme assay. The hyaluronidase inhibitory assay was conducted following the method described by Sahasrabudhe and Deodhar [34] with few modifications. Each 12.5 µL of 8 mg/mL hyaluronidase in 0.1 M acetate buffer (pH 3.6) and the test sample were mixed and incubated at 37 °C for 20 min. The mixture was treated with 25 µL of 12.5 mM CaCl_2_ and incubated again at 37 °C for 20 min. After incubation, 62.5 µL of 2.4 µg/mL hyaluronic acid in 0.1 M acetate buffer (pH 3.6) was added and incubated at 37 °C for 40 min.

Next, the mixture reaction developed the color upon adding 2 μL of 0.4 N sodium hydroxide (NaOH) and 20 μL of 0.4 N potassium tetraborate tetra-hydrate, followed by incubation in the water bath at 100 °C for 3 min. DMAB solution containing 0.4 g of DMAB dissolved in 35 mL of 100% acetic acid and 5 mL of 10N hydrochloric acid (HCl) was prepared. Finally, 600 μL of DMAB was added to the mixture solution after cooling to room temperature (22 ± 1 °C) and incubated at 37 °C for 20 min. The absorbance was measured at a wavelength of 585 nm. The IC_50_ values were calculated by nonlinear regression using PRISM version 8.4.3.

### 4.7. Molecular Composition Analysis by Gel Permeation Chromatography (GPC)

The weight-average molecular mass (Mw) and polydispersity (Mw/Mn) of PHPs were measured via gel permeation chromatography (GPC) using a Waters e2695 instrument with Ultrahydrogel 120, 250, 500, 1000 (Waters, Milford, MA, USA) column (7.8 mm × 300 mm) and a refractive index detector (RID). Then, 5 mg of freeze-dried PHP and CHP powder were dissolved in 1 mL of distilled water and filtered through a 0.45 µm filter, followed by injection of 100 µL of PHPs and CHP at 35 °C. The mobile phase was 0.1 M NaNO_3_ at a flow rate of 1 mL/min, and 0.3 mg/mL of pullulan was used as the standard. A calibration curve of standard was plotted as the logarithm of relative molecular weight (Mw, Da) versus the retention time (t, min), the regression equation was obtained, and the Mw of PAPs and CHP was determined based on the retention time of polysaccharide solution. The data were processed using Empower 3 Chromatography Data Software (Waters).

## 5. Conclusions

In conclusion, a recombinant β-agarase (rGaa16Bc) from *Gilvimarinus agarilyticus* JEA5 was cloned, and its biochemical properties were analyzed. Moreover, we examined the hyaluronidase inhibition activity of the product partially hydrolyzed by rGaa16Bc. This is the first report of the hyaluronidase inhibition of polysaccharides partially hydrolyzed by agarose. Gaa16B has a GH16 family domain as well as a carbohydrate-binding domain, both of which are typical features of GH16 β-agarases. The Gaa16B catalytic domain showed high activity at elevated temperatures and within a relatively wide pH range. It also has good thermostability and a significantly high *V*_max_ value compared to previously reported agarases. These characteristics suggest that Gaa16B is a good candidate for industrial applications in the cosmetic, pharmaceutical, and food industries. Furthermore, the results of the hyaluronidase inhibition assay provide sufficient information to justify further investigation on new applications of the partial hydrolysis product of agarases.

## Figures and Tables

**Table 1:** Pairwise alignment analysis and comparison of the deduced amino acid sequence of Gaa16B with other β-agarases.

	Identity (%)	Similarity (%)	Gap (%)	Accession No.	Remark
*Gilvimarinus polysaccharolyticus*	90.7	93.8	2.7	WP_049721028.1	Uncharacterized
*Gilvimarinus chinensis*	89.7	93.5	2.2	WP_020208740.1	Uncharacterized
*Microbulbifer thermotolerans*	75.1	85.8	5.4	WP_067155675.1	Uncharacterized
*Saccharophagus degradans* 2-40	55.9	70.9	4.2	ABD80437.1	Characterized
*Pseudomonas* sp. ND137	43.5	55.5	29.0	BAD88713.1	Characterized
*Microbulbifer elongatus*	37.8	50.5	28.8	BAC99022.1	Characterized

**Table 2:** Gel permeation chromatography of PHPs and CHP.

Sample Name	MP (Daltons)	Mw (Daltons)	Polydispersity	% Area
PHP5	22,686	102,206	5.520	70.01
PHP10	16,636	115,319	6.141	71.13
PHP20	14,948	109,822	5.843	69.12
PHP30	14,109	92,705	5.761	66.81
PHP60	10,635	88,617	6.494	59.85
CHP	459	473	1.034	84.28

MP: molecular weight of the peak maximum, Mw: average molecular weight of the peak.

**Table 3:** Hyaluronidase inhibition rate (%) of tested samples at concentration of 1 mg/mL and IC_50_.

Sample Name	Sample Solution (mg/mL)	Inhibition (%)	IC_50_ (mg/mL)
PHP5	1	63.94 ± 0.27	0.47 ± 0.04
PHP10	1	64.02 ± 0.36	0.40 ± 0.02
PHP20	1	63.65 ± 0.28	0.31 ± 0.05
PHP30	1	62.81 ± 0.50	0.41 ± 0.03
PHP60	1	63.29 ± 0.39	0.57 ± 0.10
CHP	1	7.46 ± 0.12	ND *
DSCG	1	65.32 ± 0.20	0.028 ± 0.01

The values are expressed as the mean ± SD in triplicate experiments. * ND: not detected.

## Data Availability

Data are contained within the article.
